# Sperm cryopreservation for impaired spermatogenesis

**DOI:** 10.1530/RAF-22-0106

**Published:** 2023-01-18

**Authors:** G Hughes, S Martins da Silva

**Affiliations:** 1 Assisted Conception Unit, Ninewells Hospital, Dundee, UK; 2 Reproductive Medicine Research Group, School of Medicine, Ninewells Hospital and Medical School, University of Dundee, Dundee, UK

**Keywords:** sperm, cryostorage, spermatogenesis, male infertility, ICSI

## Abstract

Sperm cryopreservation for men with severely impaired spermatogenesis is one of the commonest reasons for short-term sperm storage, usually in advance of fertility treatment. Cryopreservation is generally very effective, although not all spermatozoa survive the process of freezing and thawing. This review considers various aspects of freezing sperm, including an overview of methods, appropriate use of cryoprotectants and practical considerations, as well as oxidative stress and mechanisms of cell cryodamage.

**Lay summary:**

Cryopreservation involves freezing of cells or tissues to preserve them for future use. Sperm cryopreservation for men with a very low sperm count is one of the commonest reasons for short-term sperm storage, usually in advance of fertility treatment. Cryopreservation is generally very effective, although not all sperm cells survive the process of freezing and thawing. This review covers various aspects of freezing sperm, including consideration of methods used and mechanisms of cell damage.

## Introduction

Cryopreserved spermatozoa are used routinely in assisted reproductive technology (ART) worldwide. The origin of sperm cryobiology spans back several centuries to 1776 when Lazzaro Spallanzani, an Italian priest and scientist, first exposed and observed human spermatozoa (known as animalcules at the time) to different temperatures. His discovery that ‘heat kills the animalcules’ was of key importance to subsequent discovery of sterilisation by Louis Pasteur. Crucially, he also reported that animalcules could survive and resume swimming after being exposed to snow and near-freezing temperatures. At the time, however, the fundamental basics of biology (sperm, egg and fertilisation) had yet to be fully understood. Almost a century later, Paolo Mantegazza published observations regarding the survival of human spermatozoa after exposure to below-freezing conditions (Mantegazza 1866). Ever the visionary, he concluded ‘If the human sperm can remain unchanged for more than 4 days at the melting ice temperature, it is certain that the science of the future will improve the breeds of horses and oxen, without forcing the enormous expense of transporting stallions and bulls, and they will be able to make artificial fertilisation with the frozen sperm, shipped at great speed from one country to another. It may also be that a husband dead on the battlefields can fertilise his wife from his cadaver and have legitimate children even after he died’.

Historical scientific advances facilitated and contributed to the evolution of cryopreservation, including the use of liquid nitrogen to freeze and preserve sperm (Hoagland & Pincus 1942) as well as studies by Parkes (Parkes 1945) who demonstrated that slow freezing was less harmful to spermatozoa than rapid cooling, concluding that human sperm should be frozen in larger-volume ampoules or tubes. But a key discovery was the use of glycerol as a cryoprotectant (Polge *et al.* 1949). As with many scientific revelations, the cryoprotective property of glycerol was discovered by chance when a series of experiments demonstrated unexpectedly high survival and motility of fowl spermatozoa after exposure to −79°C. Addition of fructose solution to spermatozoa was usual practice at the time, to achieve partial dehydration prior to freezing. A bottle kept in the laboratory fridge containing 10% glycerol and 1% albumin (Meyer's solution, used for fixing smears before staining) had been incorrectly labelled as fructose and was used in the experiments by error. Nonetheless, the fortuitous discovery that glycerol and other agents could be used as cryoprotectant(s) to significantly increase spermatozoa survival was fundamental to further development and clinical translation of sperm cryobiology. The first calf born from frozen-thawed sperm by artificial insemination was subsequently reported in 1952 (Polge 1952) and the use of frozen-thawed sperm from a person who was dead at the time of inseminating his wife was reported shortly after, in 1953 (Bunge & Sherman 1953).

Mainstream cryopreservation of human spermatozoa followed agricultural application and was first introduced in the 1960s (Sherman 1973). The inaugural human sperm banks were in Iowa, USA and Tokyo, Japan (Park 2018). However, fertility treatment using donor sperm led to public outcry, levelling accusations of adultery and creation of illegitimate children, as well as political and religious objections, so clinics redirected their focus to fertility preservation for men. Political and sociocultural change in the 1970s facilitated subsequent acceptance of donor insemination; however, most treatment at that time used freshly donated unprepared semen. Development of semen processing techniques, either by swim up or density-gradient centrifugation (DGC), was a collateral benefit from the development of IVF, but it was the discovery (circa 1984) that semen could transmit the HIV virus (Stewart *et al.* 1985) and the introduction of statutory quarantine requirements with restrictions on the use of fresh semen that helped sperm banks finally gain acceptance. Fertility treatment using screened quarantined cryopreserved donor sperm is now widely available and highly commercialised. The human sperm bank market is valued at approximately $4.33 billion (2019) and is expected to reach around $5.45 billion by 2026, expanding at a compound annual growth rate of 3.3% (https://dataintelo.com/report/sperm-bank-market/ ).

### Autologous sperm cryopreservation

Beyond sperm donor programmes, sperm banking is of fundamental importance because it allows cryopreservation and autologous use of gametes at a later date. It is therefore highly relevant to patients facing gonadotoxic treatment. Improvements in cancer detection and treatment have resulted in prolonged survival and cure of patients (Dal Maso *et al.* 2019). As such, the emphasis of cancer care is not solely focussed on survival and has shifted to include consideration of quality of life, including fertility for men (and women) of reproductive age. The negative effect of cancer and its treatment on spermatogenesis is well established (Meistrich 2013). Although a large proportion of men undertaking cancer treatment retain or regain spermatogenesis and achieve natural conception (Brydoy *et al.* 2005), sperm cryopreservation is undoubtedly the best pre-treatment insurance, offering an opportunity for future fertility. But sperm cryopreservation has a reach beyond orchidectomy, chemotherapy, and/or radiotherapy as it also offers reproductive potential for transitioning transwomen as well as those undertaking treatment for benign medical conditions where treatment is likely to affect spermatogenesis and/or reproductive function (Rozati *et al.* 2017).

**Figure 1:**
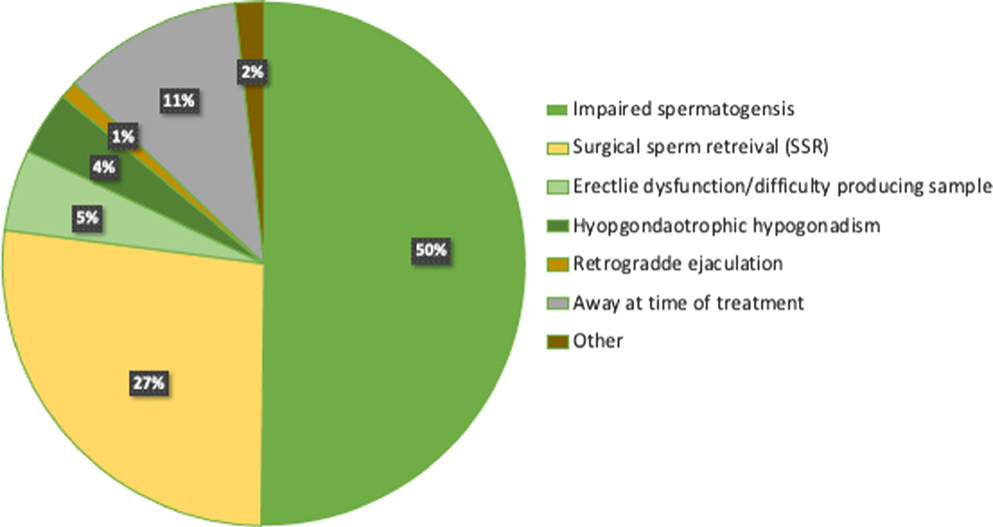
Audit of sperm samples cryostored at Ninewells Assisted Conception Unit, Dundee, excluding samples in long-term storage for fertility preservation (*n* = 279). 50% of samples were stored due to significantly impaired spermatogenesis (*n* = 140).

**Figure 2:**
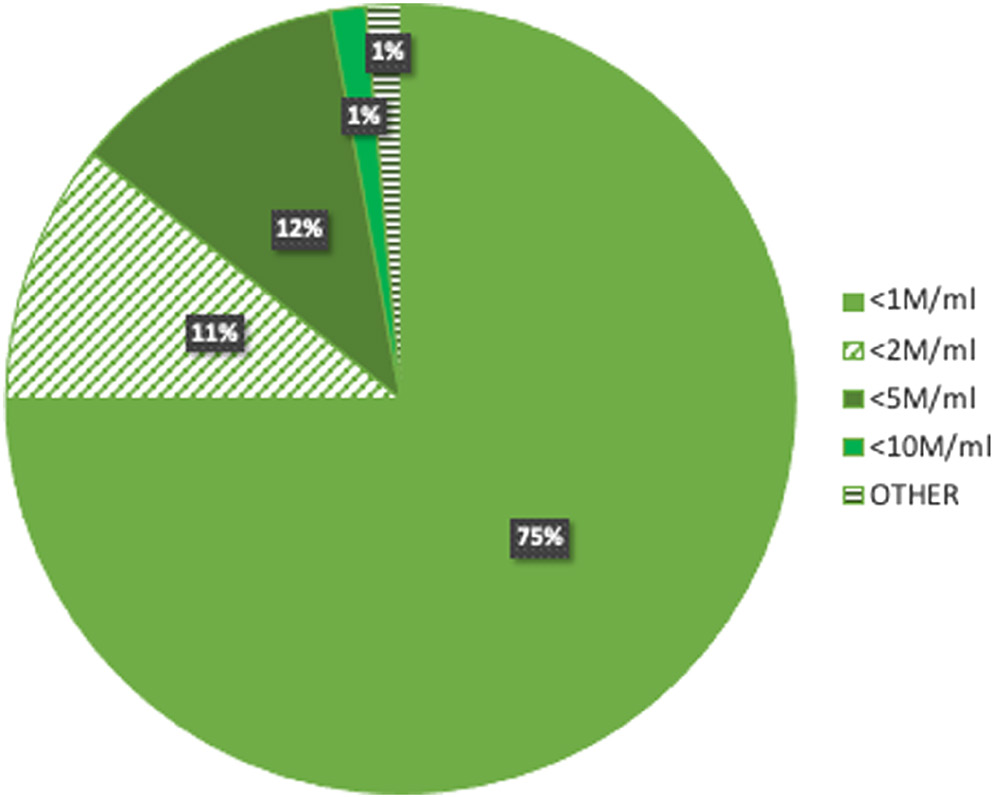
Sperm samples cryostored as treatment back up at Ninewells Assisted Conception Unit, Dundee (*n* = 140). All samples cryostored due to varying degrees of significantly impaired spermatogenesis. The majority of samples were stored where sperm concentration <1 million/mL (75%; *n* = 105).



Sperm cryopreservation for men with severely impaired spermatogenesis of both known and unexplained aetiology, usually in advance of ART, is arguably the commonest reason for short-term freezing sperm. Indeed, a recent audit of sperm cryostored at Ninewells Assisted Conception Unit, Dundee (*n* = 279; excluding samples in long-term storage for fertility preservation), found that 50% of samples stored were from men with severe oligozoospermia, as a precaution in case of azoospermia on the day of egg collection. Of the remaining samples, 27% (*n* = 75) were stored following surgical sperm retrieval (SSR), 11% (*n* = 31) cryostored samples were from those likely to be away at the time of treatment including working offshore or military deployment, 5% (*n* = 14) samples stored were due to erectile dysfunction (ED) or where difficulties had been previously encountered or were anticipated in producing sperm on the day of egg collection, 4% (*n* = 11) samples were stored following medical induction of spermatogenesis for hypogonadotrophic hypogonadism or pituitary dysfunction, and 3% (*n* = 5) samples were stored for other reasons, including quarantine prior to surrogacy (Fig. 1). Clearly, an uncertain prognosis for ongoing sperm production represents no pressing need for fertility preservation, yet cryopreservation prior to ART offers reassurance for those with severely impaired spermatogenesis in case of azoospermia on the day of egg collection. A retrospective study of 247 men who undertook sperm cryopreservation for impaired spermatogenesis prior to assisted conception reported 52% (34/66) risk of azoospermia in at least one ICSI attempt when at least one total sperm count was <100,000 vs 3% (5/181) when all counts were ≥100,000 (*P* < 0.0001) (Montagut *et al.* 2015). Nonetheless, who and when to offer autologous sperm cryopreservation is not clearly defined and therefore practices vary. For example, sperm cryopreservation is offered to those with severely impaired spermatogenesis in our centre, with the majority of samples stored for those with sperm concentration <1 million/mL (Fig. 2). However, although sperm cryostorage as back up for ICSI may be justified, it is worth noting that post-thaw samples are likely to have significantly lower sperm count and poorer total and progressive motility (Petyim *et al.* 2014). Cryosurvival factor (CSF) is calculated using the formula:

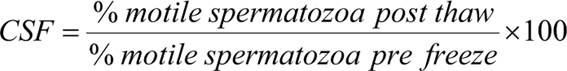



Despite numerous achievements in sperm cryobiology and widespread clinical use of cryopreserved sperm, the reality is that not all spermatozoa survive the freezing and thawing process, with post-thaw sperm concentration and motility up to 50% lower than respective pre-freeze values in humans (Mortimer *et al.* 2022). Various factors during the freezing process contribute to this phenomenon, mostly driven, either directly or indirectly, by ice formation and osmotic stress.

### Freezing sperm

The main objective of freezing semen is to maintain sperm viability and function over an extended period of time. Given that all metabolic reactions and biological processes are suspended, cryopreserved sperm can theoretically be kept indefinitely. This is supported by reports of pregnancies following ART using semen frozen for at least several decades in both humans (Horne *et al.* 2004, Feldschuh *et al.* 2005, Szell *et al.* 2013) and farm animals (Salamon & Maxwell 2000). However, semen is very heterogeneous and successful cryopreservation varies between species, between individuals of the same species, and even between ejaculates from the same individual.

Liquid nitrogen (LN_2_) is an inert cryogenic fluid. All biological activity stops below the glass transition temperature of water (around −80 to −135°C) (Bojic *et al.* 2021). Spermatozoa are routinely cryostored at −196°C in liquid nitrogen or vapour phase. However, human cells usually operate within an extremely narrow temperature range (36–38°C), and unprotected cooling and thawing of cells are not compatible with viability. Spermatozoa are very small cells with a relatively large surface area and potentially less susceptible to cryodamage compared to larger cells. Nonetheless, the ability to induce and reverse low-temperature states in a controlled manner with minimal transition-related damage and destruction, specifically cold shock and chill damage above 0°C and intracellular ice formation as the temperature falls below freezing to −130°C, is critical to successful cryostorage and subsequent revival of cells. In practice, this is the central challenge of cryopreservation. To mitigate these effects, two protective actions must be carried out: selection of an appropriate cooling (and thawing) rate and use of cryoprotectant(s).

### Slow freezing

**Figure 3:**
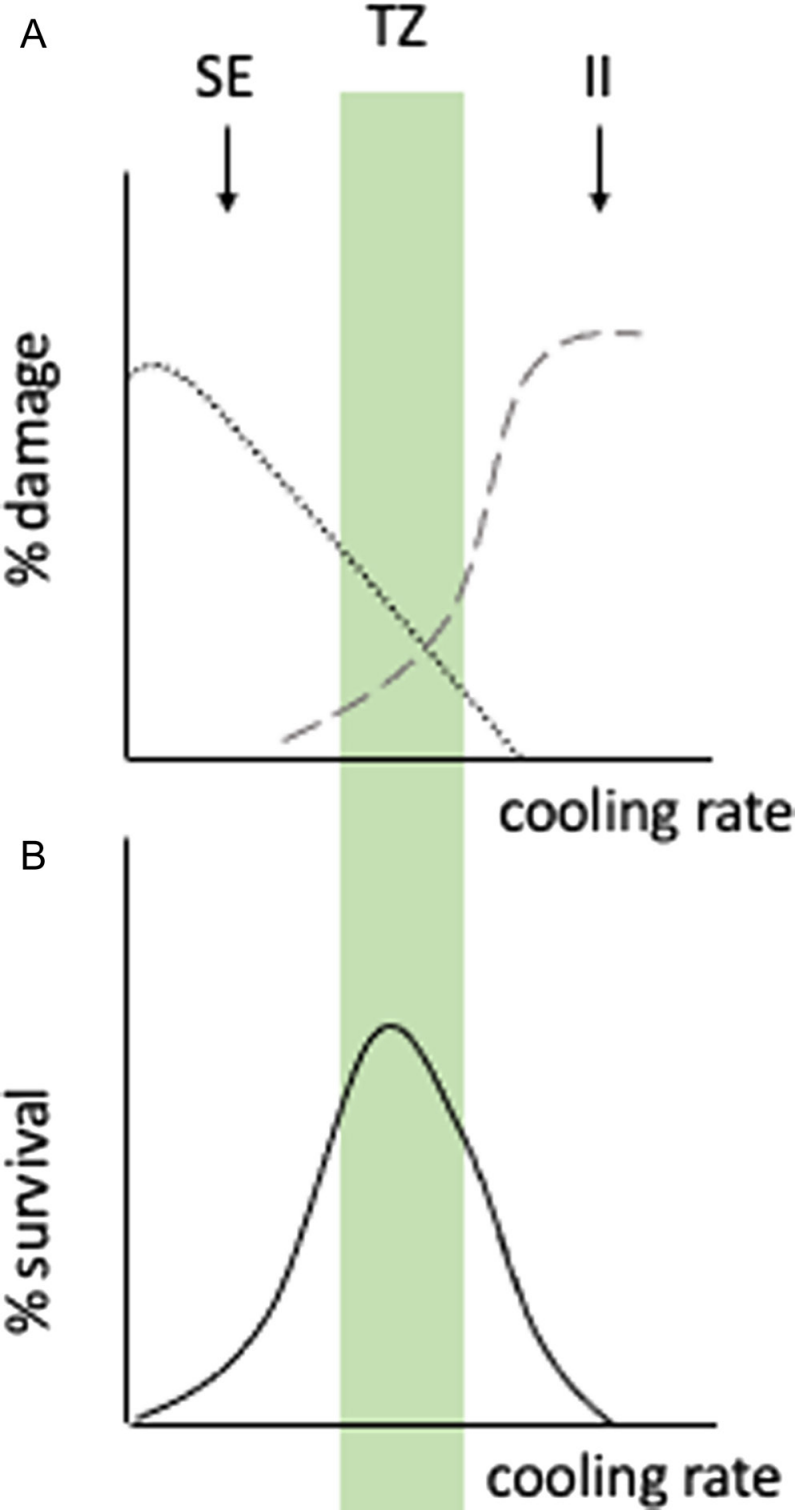
Cryodamage and cell survival during cryopreservation. (A) High cell damage due to solution effect (SE) when cooling rate is slow (dotted line). High cell damage due to intracellular ice (II) when cooling rate is high (dashed line). (B) Overall cell survival (solid line). Transition zone (TZ), where negative effects of II and SE are minimised, describes optimal cooling conditions for cryopreservation (green-shaded area).

Cryopreservation inflicts cellular damage largely as a result of uncontrolled transition between normal and low-temperature states, specifically mechanical damage from dehydration and shrinkage of cells during freezing and/or rehydration and swelling upon thawing. Importantly, cell survival is influenced by both formation of intracellular ice and osmotic stress. If a sample is cooled too quickly, then spermatozoa do not efficiently dehydrate and formation of intracellular ice results in membrane disruption and cell death. If cooling is too slow, then osmotic stress arises from solution effect, due to the interaction of membrane proteins and high ion concentrations extracellularly and/or mechanical disruption of the cell membrane due to low cell volume. A transition zone (TZ), where negative effects of both intracellular ice (II) and solution effect (SE) are minimised, describes optimal cooling conditions for cryopreservation (Fig. 3). This varies for different cells and different species. Conventional (slow) freezing is the usual approach for human sperm, where cooling to −196°C occurs over several hours by stepwise reduction in external temperature conditions (ideally achieving an initial cooling rate of 0.5–1°C/min to 5°C and then 10°C/min) (Whaley *et al.* 2021).

### Cryoprotectant agents

The addition of cryoprotectant agents (CPAs) to cryopreservation medium (CPM) aims to protect spermatozoa against both mechanical and ionic effects of dehydration and ice crystal formation, as well as stabilising the cell membrane (Elliott *et al.* 2017). CPAs may be permeating (low-molecular-weight compounds that pass through the cell, e.g. glycerol, dimethyl sulfoxide (DMSO), ethylene glycol, propylene glycol) or non-permeating (higher-molecular-weight compounds that generally act to promote fast cellular dehydration, e.g. sucrose, polyvinylpyrrolidone (PVP), raffinose, and trehalose). Permeating and non-permeating CPAs are usually used in combination. However, caution is required as permeating CPAs may be toxic and result in cell damage and death. The addition of CPAs also exerts osmotic stress by increasing the osmolarity of the surrounding medium, resulting in sequential cell shrinkage and then swelling during permeation. Most protocols therefore require slow (dropwise) addition of CPM with continual mixing.

Glycerol is the commonest permeating CPA in commercially available CPM, usually in combination with sucrose or glucose as non-permeating CPA (Raad *et al.* 2018). CPM also contains other agent(s) to stabilise the plasma membrane, for example egg yolk, albumin, milk, or glycine, which either alter the lipid composition of the cell membrane or directly interact with membrane lipids and proteins. Chelators, for example ethylenediaminetetraacetic acid (EDTA) and citrate, are included to stabilise intracellular calcium concentration, as well as compounds to inhibit lipid peroxidation and protect against oxidative stress. Lastly, CPM contains buffers to maintain pH stability, usually zwitterionic buffers, for example HEPES or TES, where the acid or base component is dipolar, as these are generally considered superior. Exact formulations of commercial CPM in clinical use are not readily available within the public domain; however, composition is highly variable (Table 1).

**Table 1:** Composition of selected commercial CPM in clinical use (2022).

	Origio sperm freezing medium	Life global sperm freezing	Sage Quinn’s advantage sperm freeze	Irvine scientific freezing medium with TYB
Glycerol	+	+	+	+
Glucose	+		+	
Sucrose	+	+	+	
Egg yolk				+ (20%)
Human serum albumin	+^†^	3.95 mg/mL	+^†^	
Synthetic serum replacement + insulin	+			
Glycine	+	+		
EDTA			+	
Sodium citrate				+
Glutamine			+	
TRIS				+
TES				+
HEPES	+	+	+	
Sodium bicarbonate	+		+	
Sodium lactate	+ (L)	+	+ (D,L)	
Calcium chloride	+		+ (dihydrate)	
Potassium chloride	+		+	
Sodium chloride	+		+	
Magnesium chloride	+			
Sodium pyruvate			+	
Sodium phosphate	+			
Potassium phosphate			+	
Magnesium sulphate			+	
Physiologic salts		+^†^		
Dextrose monohydrate		+		
Fructose				+
Gentamicin	+		+	+
Phenol red			+	

^†^Exact amount/composition not specified by the manufacturer.

### Sperm cryodamage and oxidative stress

Although multifactorial, oxidative stress (OS) is an important mechanism underlying human sperm cryodamage. Induction of OS results from increased generation of reactive oxygen species (ROS) and nitrogen reactive species (RNS). Importantly, ROS such as hydrogen peroxide (H_2_O_2_), superoxide anions (O^2–^) and hydroxyl radicals (OH^–^) can cause lipid peroxidation of the cell membrane and disruption of mitochondria, sperm DNA damage as well as apoptosis and cell death (Martins da Silva 2019). Spermatozoa are particularly susceptible to ROS-induced oxidation due to the abundance of polyunsaturated fats in the plasma membrane (Aitken *et al.* 2014). Lipid peroxidation alters cell membrane fluidity and permeability with loss of membrane integrity, as well as detrimental effects on motility (Baker *et al.* 2015, Nowicka-Bauer *et al.* 2018). Cellular structure damage, including the acrosome and plasma membrane, may also subsequently manifest as reduced ability to fertilise an egg (Bollwein *et al.* 2008). This may have implications for downstream clinical treatment, notably requiring the use of ICSI rather than IVF or intrauterine insemination (IUI) for some patients. Many studies have investigated the effects of supplementing CPM with antioxidants to neutralise ROS, both to minimise DNA damage and improve quality and/or preserve function of post-thaw spermatozoa. Both synthetic and natural antioxidant compounds have been added to CPM, with some showing noticeable and positive effects on semen characteristics both* in vitro* and* in vivo* (Amidi *et al.* 2016, Hezavehei *et al.* 2018). Among others, myo-inositol is one of the most powerful naturally occurring antioxidants that has been reported to be effective in improving sperm quality and motility when used both* in vivo* (Montanino Oliva *et al.* 2020, Santoro *et al.* 2021) and* in vitro* (Artini *et al.* 2017, Ghasemi *et al.* 2019). Myo-inositol appears to be helpful in protecting against OS during cryopreservation (Azizi *et al.* 2022) and improves sperm characteristics (vitality and motility) when added after thawing (*P*  <0.05) (Ponchia *et al.* 2021). Antioxidants can be divided into two categories (enzymatic and non-enzymatic) according to chemical structure. Enzymatic antioxidants include glutathione peroxidase (GPx), catalase and superoxide dismutase (SOD). They work by breaking down and removing free radicals, in the presence of co-factors such as copper, zinc, manganese and iron. Non-enzymatic antioxidants work by interrupting free radical chain reactions and include vitamin C, vitamin E, carotenoids, glutathione, taurine and melatonin as well as co-factors selenium and zinc. A recent systematic review and meta-analysis showed overall positive effect of antioxidants on progressive (but not total) sperm motility and viability, as well as a reduction in DNA damage and H_2_O_2_ production during the freeze-thaw process (Bahmyari *et al.* 2020). However, while data support addition of antioxidants to CPM, they do not commonly feature in commercial preparations.

ROS production is associated with damage to sperm DNA and chromatin structure as well as alterations to DNA methylation (Khosravizadeh *et al.* 2022). Notably, spermatozoa from infertile men appear to be more vulnerable to cryodamage compared to gametes from fertile men (Donnelly *et al.* 2001). Yet, the wider implications of cryopreservation on sperm quality and function are not completely understood. Cryopreservation has been reported to affect the expression of key genes related to fertility potential, specifically *SNORD116, PWSAS* and *UBE3A*, which may have implications for offspring conceived using cryopreserved sperm (Valcarce *et al.* 2013). Gene and protein expression, mRNA stability and epigenetic content of spermatozoa may be affected by the freeze-thaw process; however, the understanding of the transgenerational effects of cryostored sperm is limited (Hezavehei *et al.* 2018).

### Cryostorage: methodology and practical aspects

Semen samples are collected by masturbation after 2–5 days of abstinence and liquefied at room temperature. Semen analysis is performed prior to processing for cryopreservation. Techniques of human sperm cryopreservation are described in detail elsewhere (Mortimer *et al.* 2022). The main debate appears to be whether to prepare samples, usually by DGC, and then freeze or to freeze unprepared samples, with subsequent preparation by DGC, which enables simultaneous removal of CPM prior to use. Freezing unprepared samples allows exploitation of the protective antioxidant properties of seminal plasma (Grizard *et al.* 1999, Donnelly *et al.* 2001) and is our preferred approach (see best practice protocol for sperm cryopreservation; Supplementary Appendix 1, see section on supplementary materials given in the end of this article). Antioxidants naturally found in semen include vitamins C, E, B9 (folic acid), trace elements zinc and selenium and micronutrients such as carnitines and carotenoids (Martins da Silva 2019), which may be beneficial given OS associated with freezing and thawing sperm (Kumar *et al.* 2019). Notably, seminal plasma total antioxidant capacity is generally lower in men with impaired spermatogenesis (Roychoudhury *et al.* 2016, Gholinezhad *et al.* 2020) so the potential advantage of freezing unprepared samples may be smaller in this patient group. Conversely, several small studies have reported higher post-thaw total and progressively motility as well as vitality for semen samples prepared prior to freezing, suggesting that minimal post-thaw processing might be advantageous, particularly for donor sperm samples where count and motility are good (Brugnon *et al.* 2013, Androni *et al.* 2021).

Use of commercially available CPM, supplements and consumables must conform to pharmaceutical standards. Medical devices require CE (Conformité Européene) marking before they can be sold in European Economic Area (EEA). CE marking signifies products that have been assessed to meet safety, health and environmental legislative requirements. This has been replaced by UK Conformity Assessed in UK and is similar to the UL mark in USA and Canada. CPM should be CE-marked or equivalent, certified as pyrogen-free and with no known sperm toxicity. UK practice requires patients to be screened for infectious diseases prior to cryostorage of gametes for autologous use (hepatitis B (HBsAg and anti-hepatitis B core), hepatitits C and HIV 1 and 2) and to complete necessary legal consents (HFEA https://portal.hfea.gov.uk/media/1756/2021-10-26-code-of-practice-2021.pdf).

Cryostorage tanks are insulated cylindrical containers made from stainless steel or aluminium. The largest high-capacity tanks can hold up to 500 L of LN_2_ and potentially thousands of samples. They usually feature temperature alarms and LN_2_ sensors and have an autofill function from secondary LN_2_ tanks when levels are detected to be low. Small tanks can hold 30–60 L of LN_2_ and are usually manual, rather than autofill. Both types of tanks have a vacuum between the outer shell and inner storage chamber, to minimise the loss of LN_2_, thus allowing the tank to maintain a nearly steady state for up to several weeks if filled properly and not opened. The inner storage chamber is suspended inside the outer shell by the neck. Cold is conducted from the bottom of the tank to the neck, so even a small amount of LN_2_ in the tank will keep the contents frozen. Because of the risk of asphyxiation when used or stored in poorly ventilated areas, LN_2_ tanks must be located in a well-ventilated area with monitored oxygen levels.

Cryopreserved specimens may be stored in either liquid or vapour phase. When submerged in LN_2_, cryopreserved samples are securely maintained at a stable temperature of −196°C. The temperature cannot rise abruptly, and therefore, there is time to rescue and relocate samples in the event of catastrophic tank failure. However, most micro-organisms, including viruses, can survive in LN_2_ (Piasecka-Serafin 1972, Bielanski *et al.* 2000). Vapour phase storage is therefore commonly used for virus-positive patient samples to prevent the risk of viral transmission by contamination of LN_2_ in direct contact between samples (Bielanski 2005). Vapour-phase nitrogen is also utilised in shipper tanks and large bulk tanks used at long-term storage repositories. The vapour in which samples are stored is typically −150°C or lower. Importantly, vapour storage tanks with an autofill function need to be continuously monitored, as a tank failure will lead to a relatively rapid rise in temperature and insufficient time for personnel to respond before gametes are compromised.

Accredited embryology and andrology laboratories that provide freezing of gametes and embryos are required to check tanks three times per week and/or have continuous monitoring via a level probe with alarms that are tested at least quarterly to ensure that cryostored samples are maintained under stringent conditions. Other essential requirements include having robust laboratory protocols, as well as personnel, knowledge and experience relating to best practice for safe and reliable cryostorage of samples (including safety data sheets and signage display), provision of appropriate training, adequate personal protective equipment (PPE) and enough LN_2_ supply for emergencies (Practice Committees of the American Society for Reproductive Medicine *et al.* 2020).

### Vitrification of sperm

Vitrification involves ultra-rapid cooling and warming rates to prevent intracellular ice formation. It is certainly the superior approach for egg and embryo cryopreservation (Cobo & Diaz 2011, Rienzi *et al.* 2017) and appears to be advantageous compared to conventional freezing methods for human sperm in terms of CSF (Li *et al.* 2019). Although the first reports of clinical outcomes using vitrified sperm were published a decade ago (Isachenko *et al.* 2012, Sanchez *et al.* 2012), vitrification remains a relatively unexplored methodology with only a few studies showing its efficacy in male gametes. Sperm vitrification appears to be simple and avoids/minimises the need for cryoprotectant (Aizpurua *et al.* 2017, Shah *et al.* 2019, Schulz *et al.* 2020). However, vitrification of sperm has limitations, not least because a very small volume is required to achieve the required cooling rate, which therefore makes cryopreservation of ejaculated semen less feasible. Single sperm vitrification has also been described (Liu & Li 2020, Maleki *et al.* 2022) and may be particularly valid for patients with significantly impaired spermatogenesis and limited numbers of spermatozoa.

One legitimate concern with vitrification methodology regards direct contact of gametes with liquid nitrogen, with inherent risk of microbial contamination. Recently, new carriers have been developed, for example SpermVD (Berkovitz *et al.* 2018), to optimise freezing protocols and control volume and speed of cooling, as well as different strategies to minimise the risk of contamination. In summary, although sperm vitrification has not yet been applied in routine sperm cryopreservation, its potential as a procedure is growing (Tao *et al.* 2020). In due course, vitrification may become a faster alternative method of sperm cryopreservation with significant benefits to fertility clinics in regard to simple equipment and easy methodology.

### Thawing sperm

Following sperm cryopreservation, a ‘test thaw’ is usually performed to assess sperm survival. The results of the test thaw should be examined prior to removing straws from cryostorage, to determine the appropriate number of straws required for treatment. For extremely poor testicular samples, the patient may benefit from the preparation of multiple straws to maximise the quality of sperm used during the ICSI procedure. Conversely, for IVF, a single straw may be sufficient for treatment providing high sperm survival. Cryoprotectant should be removed quickly when thawing sperm (see best practice protocol for sperm thawing; Supplementary Appendix 2). This is typically achieved by diluting the sample with culture media. However, there is a significant risk of cell damage and death due to uncontrolled water diffusion and osmotic shock if a large volume of media is added. As such, media should be added to the frozen-thawed sample drop-by-drop, with the aim of gradually establishing an osmotic equilibrium between the intracellular cytosol of the spermatozoa and the extracellular environment. Samples are then washed by centrifugation. When preparing frozen-thawed ejaculates with a low spermatozoa concentration, the samples should be washed at a relatively high speed (800 ***g***) to maximise the concentration of spermatozoa in the final preparation. Conversely, testicular sperm are fragile, and the sample should be centrifuged at a low speed (≤200 ***g***) to prevent damage.

### How long should we freeze sperm?

Recent UK legislative changes allow sperm storage for up to 55 years. While long-term storage of sperm has no impact on clinical outcomes (Huang *et al.* 2019), utilisation of cryostored sperm is notoriously low, although relatively few studies have reported this. A 15-year cryopreservation program involving 776 men with a diagnosis of malignancy who were referred for semen cryopreservation before proceeding with chemotherapy and/or radiotherapy reported 5.2% utilisation (Ragni *et al.* 2003); epidemiological analysis of sperm cryopreservation for male patients with cancer at the University of Pennsylvania revealed less than 5% of patients utilised their specimens for reproductive purposes (Chung *et al.* 2004); an evaluation of sperm cryopreservation services for cancer patients found that 4% patients utilised their specimens for reproductive purposes (Chang *et al.* 2006). These three studies were from European, American and Asian centres; yet findings are very comparable. Similarly, a retrospective study of 545 patients suffering from cancer or benign diseases over a decade (January 2008 and July 2018) reported 5.3% utilisation (Vomstein *et al.* 2021). Notably, a retrospective review of 1442 sperm samples cryopreserved for fertility treatment (IUI, IVF/ICSI) as well as fertility preservation related to cancer treatment or military deployment reported overall utilisation rates of 19.3% (Machen *et al.* 2018). This study population is arguably much more representative of all those presenting for sperm cryostorage but again highlights the disparity between numbers of samples cryostored and those used. Clinical provision of sperm cryopreservation for impaired spermatogenesis thus raises significant practical challenges in terms of space and facilities required, as well as costs for monitoring and audit as well as administrative oversight.

## Conclusions and future perspectives

Sperm cryopreservation for impaired spermatogenesis is an important, and effective, part of fertility management. Nonetheless, cryodamage following slow freezing has detrimental effects on sperm quality and function post-thaw. We need better understanding of the cellular and molecular changes that occur during freezing and the mechanisms resulting in cryo-injury, to optimise sperm cryopreservation. Alternatively, sperm vitrification may offer a better option, particularly for men with significantly impaired spermatogenesis. Although cryopreserved spermatozoa have been used in fertility treatment for decades, transgenerational effects are uncertain and long-term follow-up studies of offspring conceived using cryopreserved spermatozoa should be performed to fully assess biological safety.

## Supplementary Material

Supplementary Appendix 1

Supplementary Appendix 2

## Declaration of interest

S Martins Da Silva is an Associate Editor of *Reproduction and Fertility*. S Martins Da Silva was not involved in the review or editorial process for this paper, on which she is listed as an author. GH declares that there is no conflict of interest.

## Funding

This work did not receive any specific grant from any funding agency in the public, commercial or not-for-profit sector.

## Author contribution statement

SMDS conceived and wrote the manuscript. GH wrote best practice protocols. GH and SMDS edited the manuscript. Both authors approved the final version.

## References

[bib1] Aitken RJ Smith TB Jobling MS Baker MA De Iuliis GN 2014 Oxidative stress and male reproductive health. Asian Journal of Andrology 16 31–38. (10.4103/1008-682X.122203) 24369131 PMC3901879

[bib2] Aizpurua J Medrano L Enciso M Sarasa J Romero A Fernandez MA Gomez-Torres MJ 2017 New permeable cryoprotectant-free vitrification method for native human sperm. Human Reproduction 32 2007–2015. (10.1093/humrep/dex281) 28938751

[bib3] Amidi F Pazhohan A Shabani Nashtaei M Khodarahmian M Nekoonam S 2016 The role of antioxidants in sperm freezing: a review. Cell Tissue Bank 17 745–756. (10.1007/s10561-016-9566-5) 27342905

[bib4] Androni DA Dodds S Tomlinson M Maalouf WE 2021 Is pre-freeze sperm preparation more advantageous than post-freeze? Reproduction and Fertility 2 17–25. (10.1530/RAF-20-0041) 35128430 PMC8812453

[bib5] Artini PG Casarosa E Carletti E Monteleone P Di Noia A Di Berardino OM 2017 In vitro effect of myo-inositol on sperm motility in normal and oligoasthenospermia patients undergoing in vitro fertilization. Gynecological Endocrinology 33 109–112. (10.1080/09513590.2016.1254179) 27908215

[bib6] Azizi M Cheraghi E Soleimani Mehranjani M 2022 Effect of myo-inositol on sperm quality and biochemical factors in cryopreserved semen of patients with asthenospermia. Andrologia 54 e14528. (10.1111/and.14528) 35841196

[bib7] Bahmyari R Zare M Sharma R Agarwal A Halvaei I 2020 The efficacy of antioxidants in sperm parameters and production of reactive oxygen species levels during the freeze-thaw process: a systematic review and meta-analysis. Andrologia 52 e13514. (10.1111/and.13514) 31967363

[bib8] Baker MA Weinberg A Hetherington L Villaverde AI Velkov T Baell J Gordon CP 2015 Defining the mechanisms by which the reactive oxygen species by-product, 4-hydroxynonenal, affects human sperm cell function. Biology of Reproduction 92 108. (10.1095/biolreprod.114.126680) 25673561

[bib9] Berkovitz A Miller N Silberman M Belenky M Itsykson P 2018 A novel solution for freezing small numbers of spermatozoa using a sperm vitrification device. Human Reproduction 33 1975–1983. (10.1093/humrep/dey304) 30285105

[bib10] Bielanski A 2005 Non-transmission of bacterial and viral microbes to embryos and semen stored in the vapour phase of liquid nitrogen in dry shippers. Cryobiology 50 206–210. (10.1016/j.cryobiol.2004.12.004) 15843010

[bib11] Bielanski A Nadin-Davis S Sapp T Lutze-Wallace C 2000 Viral contamination of embryos cryopreserved in liquid nitrogen. Cryobiology 40 110–116. (10.1006/cryo.1999.2227) 10788310

[bib12] Bojic S Murray A Bentley BL Spindler R Pawlik P Cordeiro JL Bauer R De Magalhaes JP 2021 Winter is coming: the future of cryopreservation. BMC Biology 19 56. (10.1186/s12915-021-00976-8) PMC7989039 33761937

[bib13] Bollwein H Fuchs I Koess C 2008 Interrelationship between plasma membrane integrity, mitochondrial membrane potential and DNA fragmentation in cryopreserved bovine spermatozoa. Reproduction in Domestic Animals 43 189–195. (10.1111/j.1439-0531.2007.00876.x) 17986172

[bib14] Brugnon F Ouchchane L Pons-Rejraji H Artonne C Farigoule M Janny L 2013 Density gradient centrifugation prior to cryopreservation and hypotaurine supplementation improve post-thaw quality of sperm from infertile men with oligoasthenoteratozoospermia. Human Reproduction 28 2045–2057. (10.1093/humrep/det253) 23760160

[bib15] Brydoy M Fossa SD Klepp O Bremnes RM Wist EA Wentzel-Larsen T Dahl O 2005 Paternity following treatment for testicular cancer. Journal of the National Cancer Institute 97 1580–1588. (10.1093/jnci/dji339) 16264178

[bib16] Bunge RG Sherman JK 1953 Fertilizing capacity of frozen human spermatozoa. Nature 172 767–768. (10.1038/172767b0) 13111181

[bib17] Chang HC Chen SC Chen J Hsieh JT 2006 Initial 10-year experience of sperm cryopreservation services for cancer patients. Journal of the Formosan Medical Association 105 1022–1026. (10.1016/S0929-6646(0960288-6) 17185246

[bib18] Chung K Irani J Knee G Efymow B Blasco L Patrizio P 2004 Sperm cryopreservation for male patients with cancer: an epidemiological analysis at the University of Pennsylvania. European Journal of Obstetrics, Gynecology, and Reproductive Biology 113(Supplement 1) S7–S11. (10.1016/j.ejogrb.2003.11.024) 15041122

[bib19] Cobo A Diaz C 2011 Clinical application of oocyte vitrification: a systematic review and meta-analysis of randomized controlled trials. Fertility and Sterility 96 277–285. (10.1016/j.fertnstert.2011.06.030) 21718983

[bib20] Dal Maso L Panato C Guzzinati S Serraino D Francisci S Botta L Capocaccia R Tavilla A Gigli A Crocetti E 2019 Prognosis and cure of long-term cancer survivors: a population-based estimation. Cancer Medicine 8 4497–4507. (10.1002/cam4.2276) 31207165 PMC6675712

[bib21] Donnelly ET Mcclure N Lewis SE 2001 Cryopreservation of human semen and prepared sperm: effects on motility parameters and DNA integrity. Fertility and Sterility 76 892–900. (10.1016/s0015-0282(0102834-5) 11704107

[bib22] Elliott GD Wang S Fuller BJ 2017 Cryoprotectants: a review of the actions and applications of cryoprotective solutes that modulate cell recovery from ultra-low temperatures. Cryobiology 76 74–91. (10.1016/j.cryobiol.2017.04.004) 28428046

[bib23] Feldschuh J Brassel J Durso N Levine A 2005 Successful sperm storage for 28 years. Fertility and Sterility 84 1017. (10.1016/j.fertnstert.2005.05.015) 16213859

[bib24] Ghasemi A Amjadi F Masoumeh Ghazi Mirsaeed S Mohammad Beigi R Ghasemi S Moradi Y Tahereh Ghazi Mirsaeed S 2019 The effect of myo-inositol on sperm parameters and pregnancy rate in oligoasthenospermic men treated with IUI: a randomized clinical trial. International Journal of Reproductive Biomedicine 17 749–756. (10.18502/ijrm.v17i10.5296) 31807723 PMC6844281

[bib25] Gholinezhad M Aliarab A Abbaszadeh-Goudarzi G Yousefnia-Pasha Y Samadaian N Rasolpour-Roshan K Aghagolzadeh-Haji H Mohammadoo-Khorasani M 2020 Nitric oxide, 8-hydroxydeoxyguanosine, and total antioxidant capacity in human seminal plasma of infertile men and their relationship with sperm parameters. Clinical and Experimental Reproductive Medicine 47 54–60. (10.5653/cerm.2020.00423) 32079054 PMC7127900

[bib26] Grizard G Chevalier V Griveau Jf Le Lannou D Boucher D 1999 Influence of seminal plasma on cryopreservation of human spermatozoa in a biological material-free medium: study of normal and low-quality semen. International Journal of Andrology 22 190–196. (10.1046/j.1365-2605.1999.00170.x) 10367240

[bib27] Hezavehei M Sharafi M Kouchesfahani HM Henkel R Agarwal A Esmaeili V Shahverdi A 2018 Sperm cryopreservation: a review on current molecular cryobiology and advanced approaches. Reproductive Biomedicine Online 37 327–339. (10.1016/j.rbmo.2018.05.012) 30143329

[bib28] Hoagland H Pincus G 1942 Revival of mammalian sperm after immersion in liquid nitrogen. Journal of General Physiology 25 337–344. (10.1085/jgp.25.3.337) 19873277 PMC2142505

[bib29] Horne G Atkinson AD Pease EH Logue JP Brison DR Lieberman BA 2004 Live birth with sperm cryopreserved for 21 years prior to cancer treatment: case report. Human Reproduction 19 1448–1449. (10.1093/humrep/deh249) 15163644

[bib30] Huang C Lei L Wu HL Gan RX Yuan XB Fan LQ Zhu WB 2019 Long-term cryostorage of semen in a human sperm bank does not affect clinical outcomes. Fertility and Sterility 112 663–669.e1. (10.1016/j.fertnstert.2019.06.008) 31371041

[bib31] Isachenko V Isachenko E Petrunkina AM Sanchez R 2012 Human spermatozoa vitrified in the absence of permeable cryoprotectants: birth of two healthy babies. Reproduction, Fertility, and Development 24 323–326. (10.1071/RD11061) 22281078

[bib32] Khosravizadeh Z Khodamoradi K Rashidi Z Jahromi M Shiri E Salehi E Talebi A 2022 Sperm cryopreservation and DNA methylation: possible implications for ART success and the health of offspring. Journal of Assisted Reproduction and Genetics 39 1815–1824. (10.1007/s10815-022-02545-6) 35713751 PMC9428082

[bib33] Kumar A Prasad JK Srivastava N Ghosh SK 2019 Strategies to minimize various stress-related freeze-thaw damages during conventional cryopreservation of mammalian spermatozoa. Biopreservation and Biobanking 17 603–612. (10.1089/bio.2019.0037) 31429586

[bib34] Li YX Zhou L Lv MQ Ge P Liu YC Zhou DX 2019 Vitrification and conventional freezing methods in sperm cryopreservation: a systematic review and meta-analysis. European Journal of Obstetrics, Gynecology, and Reproductive Biology 233 84–92. (10.1016/j.ejogrb.2018.11.028) 30580229

[bib35] Liu S Li F 2020 Cryopreservation of single-sperm: where are we today? Reproductive Biology and Endocrinology: RB&E 18 41. (10.1186/s12958-020-00607-x) PMC7216378 32398019

[bib36] Machen GL Harris SE Bird ET Brown ML Ingalsbe DA East MM Reyes M Kuehl TJ 2018 Utilization of cryopreserved sperm cells based on the indication for storage. Investigative and Clinical Urology 59 177–181. (10.4111/icu.2018.59.3.177) 29744474 PMC5934279

[bib37] Maleki B Khalili MA Gholizadeh L Mangoli E Agha-Rahimi A 2022 Single sperm vitrification with permeable cryoprotectant-free medium is more effective in patients with severe oligozoospermia and azoospermia. Cryobiology 104 15–22. (10.1016/j.cryobiol.2021.11.176) 34822804

[bib38] Mantegazza P 1866. Rentic. Reale Instit. Lomb 3 183.

[bib39] Martins da Silva SJ 2019 Male infertility and antioxidants: one small step for man, no giant leap for andrology? Reproductive Biomedicine Online 39 879–883. (10.1016/j.rbmo.2019.08.008) 31727498

[bib40] Meistrich ML 2013 Effects of chemotherapy and radiotherapy on spermatogenesis in humans. Fertility and Sterility 100 1180–1186. (10.1016/j.fertnstert.2013.08.010) 24012199 PMC3826884

[bib41] Montagut M Gatimel N Bourdet-Loubere S Daudin M Bujan L Mieusset R Isus F Parinaud J Leandri R 2015 Sperm freezing to address the risk of azoospermia on the day of ICSI. Human Reproduction 30 2486–2492. (10.1093/humrep/dev234) 26364079

[bib42] Montanino Oliva M Buonomo G Carra MC Lippa A Lisi F 2020 myo-inositol impact on sperm motility in vagina and evaluation of its effects on foetal development. European Review for Medical and Pharmacological Sciences 24 2704–2709. (10.26355/eurrev_202003_20540) 32196621

[bib43] Mortimer D Björndahl L Barratt CLR Castilla JA Menkveld R 2022 A practical guide to basic laboratory andrology. New York, NY: Cambridge University Press. (10.1017/9781009181648)

[bib44] Nowicka-Bauer K Lepczynski A Ozgo M Kamieniczna M Fraczek M Stanski L Olszewska M Malcher A Skrzypczak W Kurpisz MK 2018 Sperm mitochondrial dysfunction and oxidative stress as possible reasons for isolated asthenozoospermia. Journal of Physiology and Pharmacology 69. (10.26402/jpp.2018.3.05) 30149371

[bib45] Park NC 2018 Sperm bank: from laboratory to patient. World Journal of Men’s Health 36 89–91. (10.5534/wjmh.182002) PMC5924960 29623696

[bib46] Parkes AS 1945 Preservation of spermatozoa at low temperatures. British Medical Journal 2 212–213. (10.1136/bmj.2.4415.212) 20786227 PMC2059602

[bib47] Petyim S Neungton C Thanaboonyawat I Laokirkkiat P Choavaratana R 2014 Sperm preparation before freezing improves sperm motility and reduces apoptosis in post-freezing-thawing sperm compared with post-thawing sperm preparation. Journal of Assisted Reproduction and Genetics 31 1673–1680. (10.1007/s10815-014-0332-y) 25212531 PMC4250457

[bib48] Piasecka-Serafin M 1972 The effect of the sediment accumulated in containers under experimental conditions on the infection of semen stored directly in liquid nitrogen (-196 degree C). Bulletin de l’Academie Polonaise des Sciences. Serie des Sciences Biologiques 20 263–267. 4554430

[bib49] Polge C 1952 Fertilizing capacity of bull spermatozoa after freezing at 79 degrees C. Nature 169 626–627. (10.1038/169626b0) 14929257

[bib50] Polge C Smith AU Parkes AS 1949 Revival of spermatozoa after vitrification and dehydration at low temperatures. Nature 164 666. (10.1038/164666a0) 18143360

[bib51] Ponchia R Bruno A Renzi A Landi C Shaba E Luongo FP Haxhiu A Artini PG Luddi A Governini L 2021 Oxidative stress measurement in frozen/thawed human sperm: the protective role of an in vitro treatment with myo-inositol. Antioxidants (Basel) 11. (10.3390/antiox11010010) PMC8773045 35052514

[bib52] **Practice Committees of the American Society for Reproductive Medicine, Society for Reproductive Biologists and Technologists, and Society for Assisted Reproductive Technology.** 2020 Cryostorage of reproductive tissues in the in vitro fertilization laboratory: a committee opinion. Fertility and Sterility 114 486–491. (10.1016/j.fertnstert.2020.06.019) 32778330

[bib53] Raad G Lteif L Lahoud R Azoury J Azoury J Tanios J Hazzouri M Azoury J 2018 Cryopreservation media differentially affect sperm motility, morphology and DNA integrity. Andrology 6 836–845. (10.1111/andr.12531) 30105872

[bib54] Ragni G Somigliana E Restelli L Salvi R Arnoldi M Paffoni A 2003 Sperm banking and rate of assisted reproduction treatment: insights from a 15-year cryopreservation program for male cancer patients. Cancer 97 1624–1629. (10.1002/cncr.11229) 12655518

[bib55] Rienzi L Gracia C Maggiulli R Labarbera AR Kaser DJ Ubaldi FM Vanderpoel S Racowsky C 2017 Oocyte, embryo and blastocyst cryopreservation in ART: systematic review and meta-analysis comparing slow-freezing versus vitrification to produce evidence for the development of global guidance. Human Reproduction Update 23 139–155. (10.1093/humupd/dmw038) 27827818 PMC5850862

[bib56] Roychoudhury S Sharma R Sikka S Agarwal A 2016 Diagnostic application of total antioxidant capacity in seminal plasma to assess oxidative stress in male factor infertility. Journal of Assisted Reproduction and Genetics 33 627–635. (10.1007/s10815-016-0677-5) 26941096 PMC4870440

[bib57] Rozati H Handley T Jayasena CN 2017 Process and pitfalls of sperm cryopreservation. Journal of Clinical Medicine 6. (10.3390/jcm6090089) PMC5615282 28925939

[bib58] Salamon S Maxwell WM 2000 Storage of ram semen. Animal Reproduction Science 62 77–111. (10.1016/s0378-4320(0000155-x) 10924821

[bib59] Sanchez R Isachenko V Petrunkina AM Risopatron J Schulz M Isachenko E 2012 Live birth after intrauterine insemination with spermatozoa from an oligoasthenozoospermic patient vitrified without permeable cryoprotectants. Journal of Andrology 33 559–562. (10.2164/jandrol.111.014274) 21868747

[bib60] Santoro M Aquila S Russo G 2021 Sperm performance in oligoasthenoteratozoospermic patients is induced by a nutraceuticals mix, containing mainly myo-inositol. Systems Biology in Reproductive Medicine 67 50–63. (10.1080/19396368.2020.1826067) 33094655

[bib61] Schulz M Risopatron J Uribe P Isachenko E Isachenko V Sanchez R 2020 Human sperm vitrification: a scientific report. Andrology 8 1642–1650. (10.1111/andr.12847) 32598551

[bib62] Shah D Rasappan S Gunasekaran K Gunasekaran K 2019 A simple method of human sperm vitrification. MethodsX 6 2198–2204. (10.1016/j.mex.2019.09.022) 31667120 PMC6812371

[bib63] Sherman JK 1973 Synopsis of the use of frozen human semen since 1964: state of the art of human semen banking. Fertility and Sterility 24 397–412. (10.1016/s0015-0282(1639678-9) 4735423

[bib64] Stewart GJ Tyler JP Cunningham AL Barr JA Driscoll GL Gold J Lamont BJ 1985 Transmission of human T-cell lymphotropic virus type III (HTLV-III) by artificial insemination by donor. Lancet 2 581–585. (10.1016/s0140-6736(8590585-9) 2863597

[bib65] Szell AZ Bierbaum RC Hazelrigg WB Chetkowski RJ 2013 Live births from frozen human semen stored for 40 years. Journal of Assisted Reproduction and Genetics 30 743–744. (10.1007/s10815-013-9998-9) 23615727 PMC3696447

[bib66] Tao Y Sanger E Saewu A Leveille MC 2020 Human sperm vitrification: the state of the art. Reproductive Biology and Endocrinology: RB&E 18 17. (10.1186/s12958-020-00580-5) PMC7060631 32145746

[bib67] Valcarce DG Carton-Garcia F Riesco MF Herraez MP Robles V 2013 Analysis of DNA damage after human sperm cryopreservation in genes crucial for fertilization and early embryo development. Andrology 1 723–730. (10.1111/j.2047-2927.2013.00116.x) 23970451

[bib68] Vomstein K Reiser E Pinggera GM Toerzsoek P Deininger S Kriesche T Biasio W Lusuardi L Toth B 2021 Sperm banking before gonadotoxic treatment: is it worth the effort? Asian Journal of Andrology 23 490–494. (10.4103/aja.aja_16_21) 33818523 PMC8451482

[bib69] Whaley D Damyar K Witek RP Mendoza A Alexander M Lakey JR 2021 Cryopreservation: an overview of principles and cell-specific considerations. Cell Transplantation 30 963689721999617. (10.1177/0963689721999617) 33757335 PMC7995302

