# Supplementary material for: Sperm cryopreservation for impaired spermatogenesis
Source: Reprod Fertil. 2023 Jan 18;4(1):e220106. doi: 10.1530/RAF-22-0106 (PMC9874962; doi:10.1530/RAF-22-0106)
Supplement: Supplementary Appendix 1 [file supplementary_table_1.pdf]

Best practice protocol for sperm cryopreservation

The same basic method of sperm cryopreservation may be used for all sperm samples regardless of source and maturity (fresh ejaculate, retrograde ejaculate, testicular spermatozoa). Ideally, cryopreservation is performed on neat semen samples. In case of very low sperm concentrations, it is advisable to concentrate the sperm before freezing. In case of very high sperm concentration, sperm can be diluted with buffer medium before freezing, if preferred.

Materials:

- Freezing tank with Liquid Nitrogen
- Class II safety cabinet
- Incubator
- Centrifuge
- Racking
- Counting chamber
- Straw Labeller
- Straw labels
- Straw sealer
- Sterile pipette tips
- Specimen pot
- Slides and coverslips
- Pipette
- Sterile long-form Pasteur pipettes
- Sample preparation and waste tubes
- Sperm straws – CBSTM high security sperm straws 0.5ml
- Sterile filling nozzle CBSTM high security straws
- Straw filling device (unit-specific)
- FertiPro SPF: SpermFreeze™

Method

1. Place the specimen pot in a class II safety cabinet and leave to liquify at room temperature for 30 minutes.
2. Assess sample volume, appearance and liquefaction (not applicable to TESE samples). Sperm concentration, motility and morphology should also be determined and recorded.
3. Cryopreservation media should be well mixed and equilibrated to room temperature before use.
4. Add the required volume of cryopreservation media according to manufacturer instructions. For example, SpermFreeze™ (FertiPro) is a ready-to-use HEPES buffered cryopreservation medium and requires 0.7ml medium for 1ml sperm.

5. Add cryopreservation media to the sperm sample in a drop-wise manner, with each drop followed by gentle agitation; this action prevents sperm damage through osmotic shock.
6. Following the addition of cryopreservation media, leave the sample to equilibrate at room temperature for 10 minutes.
7. Following equilibration, aspirate (using syringe and adapter or aspirator) the sperm/cryopreservation media mixture into a CBSTM high security sperm straw until the sperm mix reaches the hydrophobic plug. This seals the straw whilst leaving an airspace in the lower part of the straw to allow for expansion during freezing. Take care to avoid contaminating the outer surface of the straw.
8. Remove the filled straw from the micro-aspirator/syringe attachment. Remove the filling nozzle. Heat-seal both ends of the CBSTM high security sperm straw using the sealer as per manufacturer's instructions and place the straw to one side within the safety cabinet. Repeat the process until all straws are filled and sealed.
9. Shake to move the air-bubble to the centre of the straw.
10. Transfer straws quickly into liquid nitrogen and store at -196 °C.
