# Supplementary material for: Sperm cryopreservation for impaired spermatogenesis
Source: Reprod Fertil. 2023 Jan 18;4(1):e220106. doi: 10.1530/RAF-22-0106 (PMC9874962; doi:10.1530/RAF-22-0106)
Supplement: Supplementary Appendix 2 [file supplementary_table_2.pdf]

Best practice protocol for sperm thawing

Materials

- Class II safety cabinet
- Centrifuge
- Racking
- Counting chamber
- Sterile pipette tips
- Slides and coverslips
- Pipette
- Sterile long-form Pasteur pipettes
- Sample preparation and waste tubes

Method

- Before use, remove as many straws or cryovials as required from the liquid nitrogen or vapour tank and place them immediately in tap water for 5 minutes (room temperature or 37 °C). Due to the large surface area-to-volume ratio of the cryopreservation straws, the contained sample will thaw rapidly
- After complete thawing, cut off the end of the straw with sterile scissors and load the insemination device (for therapeutic use) or expel the contents to determine post-thaw motility (to check the freezing process). The straw contents are emptied into a sterile tube by cutting below the hydrophobic bung at the top of the straw with a pair of sterile scissors. Any residual sample remaining in the straw is used to create an examination slide for assessing semen parameters.
- Remove the cryoprotectant by adding culture medium before centrifugation for 10 minutes at 500g. Remove the supernatant and dilute the sperm pellet in culture medium to the appropriate volume.
- If necessary, use sperm preparation techniques after thawing the semen to eliminate dead sperm cells and debris. Dilute the concentrated sperm in a suitable insemination medium
